# Crystal structure of dicarbon­yl[μ_2_-methyl­enebis(di­phenyl­phosphane)-κ^2^ *P*:*P*′][μ_2_-2-(2,4,5-tri­methyl­phen­yl)-3-oxoprop-1-ene-1,3-di­yl](tri­phenyl­phosphane-κ*P*)ironplatinum(*Fe*—*Pt*)–di­chloro­methane–toluene (1/1/2), [(OC)_2_Fe(μ-dppm)(μ-C(=O)C(2,4,5-C_6_H_2_Me_3_)=CH)Pt(PPh_3_)]

**DOI:** 10.1107/S2056989019015573

**Published:** 2019-11-22

**Authors:** Lukas Brieger, Isabelle Jourdain, Michael Knorr, Carsten Strohmann

**Affiliations:** a Technical University Dortmund, Chemistry and Chemical Biology, Inorganic Chemistry, Otto-Hahn Str. 6, 44227 Dortmund, Germany; b Institut UTINAM UMR 6213 CNRS, Université Bourgogne Franche-Comté, 16, Route de Gray, 25030 Besançon Cedex, France

**Keywords:** crystal structure, terminal alkyne, iron, platinum, heterobimetallic, metal–metal bond dimetalla­cyclo­pentenone, bis­(di­phenyl­phosphino)methane, hydrogen bonding, C—H⋯π inter­actions

## Abstract

The title compound, [FePt(C_12_H_12_O)(C_18_H_15_P)(C_25_H_22_P_2_)(CO)_2_]·2C_7_H_8_·CH_2_Cl_2_ or [(OC)_2_Fe(μ-dppm)(μ-C(=O)C(2,4,5-C_6_H_2_Me_3_)=CH)Pt(PPh_3_)], represents an example of a diphosphane-bridged heterobimetallic dimetalla­cyclo­pentenone complex resulting from a bimetallic activation of 1-ethynyl-2,4,5-tri­methyl­benzene and a metal-coordinated carbonyl ligand. The bridging μ_2_-C(=O)C(2,4,5*-*C_6_H_2_Me_3_)=CH unit (stemming from a carbon–carbon coupling reaction between CO and the terminal alkyne) forms a five-membered dimetalla­cyclo­pentenone ring, in which the C=C bond is π-coordinated to the Fe centre. The latter is connected to the Pt centre through a short metal–metal bond of 2.5770 (5) Å. In the crystal, the complex is solvated by one di­chloro­methane and two toluene mol­ecules.

## Abstract 2

The title compound, [(OC)_2_Fe(μ-dppm)(μ-C(=O)C(2,4,5-C_6_H_2_Me_3_)= CH)Pt(PPh_3_)], represents an example of a diphosphane-bridged heterobimetallic dimetalla­cyclo­pentenone complex resulting from a bimetallic activation of 1-ethynyl-2,4,5-tri­methyl­benzene and a metal-coordinated carbonyl ligand.

## Chemical context   

The coordination and transformation of alkynes on homobimetallic transition-metal complexes, in which the two metal centres are in close contact *via* a metal–metal bond, has been investigated intensively (Liddle, 2015 ▸). For example, during the course of a Pauson–Khand reaction, an acetyl­enic triple bond is added across [Co_2_(CO)_8_)], yielding a dimetalla­tetra­hedrane [Co_2_(CO)_6_(μ-C_2_ *RR*′)] (Bennett *et al.*, 1992 ▸; Clément *et al.*, 2007 ▸).

The activation of alkynes by heterodinuclear transition-metal complexes *L_n_M*—*M*′*L_n_* has also stimulated much inter­est because of possible synergic effects exerted by the close proximity of metal centres (with different coordination spheres, oxidation states, …; Stephan, 1989 ▸; Ritleng & Chetcuti, 2007 ▸; Cooper *et al.*, 2012 ▸). Among the different heterobimetallic combinations, the investigation of the group 8–10 Fe–Pt couple has been pioneered by Fontaine *et al.* (1988 ▸), who has shown that, upon treatment of the μ-carbonyl complex [(OC)_3_Fe(μ-dppm)(μ-CO)Pt(PPh_3_)] (dppm = bis(di­phenyl­phosphino)methane) with *Ar*C≡CH (*Ar* = Ph, *p*-Tol), dimetalla­cyclo­pentone complexes are formed, stemming from a carbon–carbon coupling reaction between CO and the alkyne. The first step involves formation of a kinetic isomer [(OC)_2_Fe(μ-dppm){μ-C(=O)C(H)=C(*Ar*)}Pt(PPh_3_)], which then evolves to the thermodynamic one [(OC)_2_Fe(μ-dppm){μ-C(=O)C(*Ar*)=C(H)}Pt(PPh_3_)]. Other strategies leading to structurally characterized dimetalla­cyclo­pentones have been reported by Yamazaki *et al.* (2005 ▸, 2006 ▸), implying the reaction of Fe(CO)_5_ with the π-alkyne-Pt^0^ complex Pt(η^2^-PhC≡CC≡CPh)(PPh_3_)_2_ or the bis-acetyl­ide-Pt^II^ compound Pt(C≡C*Tp*)_2_(dppe) (*Tp* = 3-thio­phene, dppe = 1,2-bis­(di­phenyl­phosphino)ethane), and leading to [(OC)_3_Fe{μ-C(=O)C(Ph)=C(C≡C-Ph)}Pt(PPh_3_)_2_] and [(OC)_2_Fe(μ-CO){μ-C(=O)C(Tp)=C(C≡C-Tp)}Pt(dppe)], respectively.

**Figure 1:**
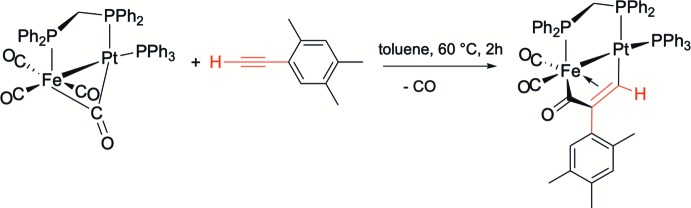
Reaction scheme for the synthesis of the title compound.

Our investigations on the reactivity of bimetallic silyl-substituted hydride complexes, [(OC)_3_Fe{Si(OMe)_3_}(μ-PPh_2_ *X*PPh_2_)Pt(H)(PPh_3_)] (*X* = CH_2_, NH), toward a huge panel of terminal aliphatic and aromatic alkynes led to σ-alkenyl complexes [(OC)_3_Fe{μ-Si(OMe)_2_(OMe)}(μ-PPh_2_ *X*PPh_2_)Pt(*R*C=CH_2_)], resulting from initial insertion into the Pt—H bond. The latter can then, depending on the function of the *R* substituent, convert to dimetalla­cyclo­pentones or to isomeric μ-vinyl­idene complexes [(OC)_3_Fe(μ-PPh_2_ *X*PPh_2_){μ-C=C(*R*)H}Pt(PPh_3_)] (Jourdain *et al.*, 2006 ▸). A third type of complex crystallographically characterized by our group is the dimetalla­cyclo­butene [(OC)_3_Fe(μ-dppm){μ-C(*o*-CF_3_-C_6_H_4_)C=C(H)}Pt(PPh_3_)]. This latter compound was obtained by treatment of [(OC)_3_Fe{Si(OMe)_3_}(μ-dppm)Pt(H)(PPh_3_)] or [(OC)_3_Fe(μ-dppm)(μ-CO)Pt(PPh_3_)] with *o*-CF_3_-C_6_H_4_C≡CH, bearing a sterically crowded –CF_3_ substituent at the *ortho*-position of the aryl group (Jourdain *et al.*, 2013 ▸). To probe whether other sterically crowded alkynes may lead to the formation of dimetalla­cyclo­butenes or rather dimetalla­cyclo­pentones, we also reacted [(OC)_3_Fe(μ-dppm)(μ-CO)Pt(PPh_3_)] with 1-ethynyl-2,4,5-tri­methyl­benzene bearing three methyl groups on the aromatic cycle; see Fig. 1 ▸.
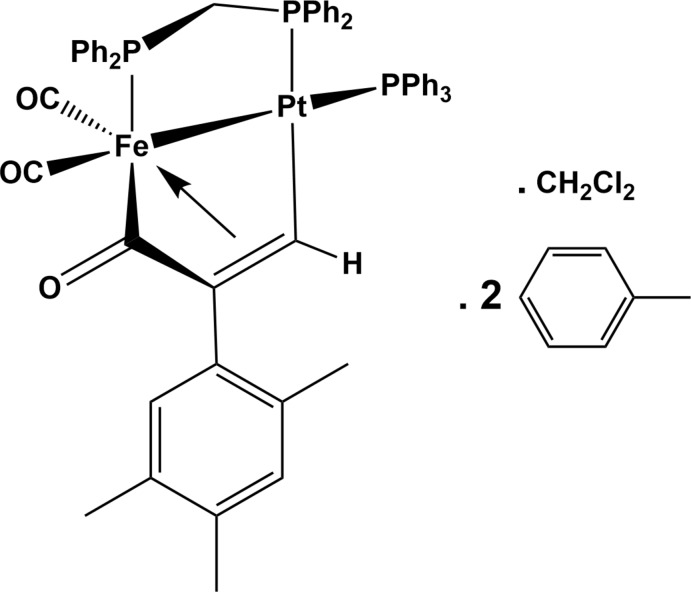


## Structural commentary   

**Figure 2:**
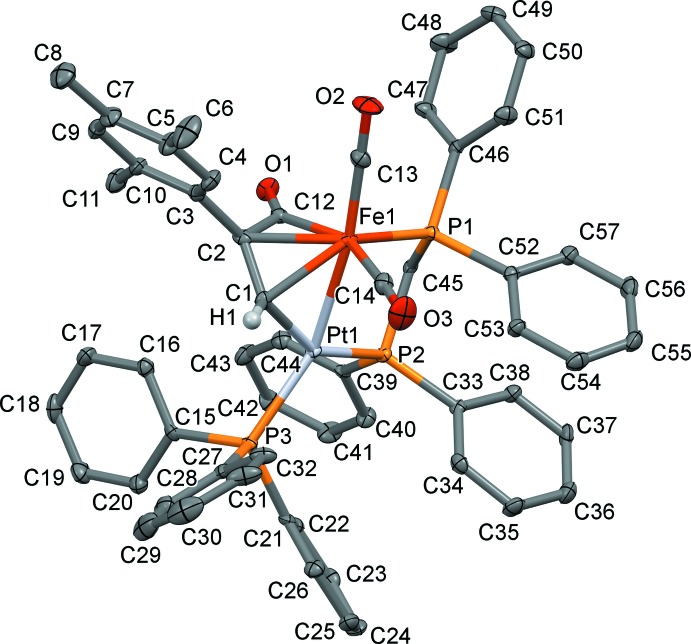
The mol­ecular structure of the title complex, with atom labelling. Displacement ellipsoids are drawn at the 30% probability level. For clarity, only H atom H1 has been included, and the solvent mol­ecules have been omitted.

The mol­ecular structure of the title heterobimetallic complex is depicted in Fig. 2 ▸. It crystallized from CH_2_Cl_2_/toluene in the monoclinic crystal system, space group *P*2_1_/*n*, together with one mol­ecule of CH_2_Cl_2_ and two mol­ecules of toluene. Selected bond lengths and bond angles are given in Table 1 ▸.

The Fe—Pt bond is bridged by a dppm ligand, forming a five-membered ring that adopts an envelope conformation, with angle P1—C45—P2 = 108.83 (17)°, and the metal–phospho­rus bonds Pt—P2 and Fe—P1 being 2.2700 (9) and 2.1857 (11) Å, respectively. These structural features are in line with those of other related structures published by our group and the Fe1—Pt1 bond length of 2.5770 (5) Å is in the range, 2.5453 (9) to 2.597 (4) Å, encountered for similar dppm-bridged compounds, *e.g.* [(OC)_2_Fe(μ-dppm){μ-C(=O)C(CH_2_)_2_=C(H)}Pt(PPh_3_)]_2_ and [(OC)_2_Fe{μ-C(=O)C(H)=C(H)}(μ-dppm)Pt(PPh_3_)] (Mohamed *et al.*, 2019 ▸; Fontaine *et al.*, 1988 ▸). When the metals are not spanned by a diphosphane ligand, the Fe—Pt bond distance is slightly longer, as in [(OC)_3_Fe{μ-C(=O)C(Ph)=C(C≡C-Ph)}Pt(PPh_3_)_2_] and [(OC)_2_Fe(μ-CO){μ-C(=O)C(Tp)=C(C≡C-Tp)}Pt(dppe)] with Fe—Pt distances of 2.608 (2) and 2.605 (2) Å, respectively (Yamazaki *et al.*, 2005 ▸, 2006 ▸). Both metals are also incorporated in a dimetalla­cyclo­pentenone unit resulting from a carbon–carbon coupling reaction between CO and the terminal alkyne giving rise to an iron-acyl group [C12—O1 = 1.207 (4) Å]. The geometry at Fe1 can be considered as distorted octa­hedral resulting from π-coord­ination of the C1=C2 bond of the five-membered [FeC(=O)C*R*=C(H)Pt] unit [C1—Fe1 = 2.107 (3) and C2—Fe1 = 2.109 (4) Å]. The C1=C2 bond length compares well with that of [(OC)_2_Fe{μ-C(=O)C(*o,p-*C_6_H_3_-F_2_)=C(H)}(μ-dppm)Pt(PPh_3_)] [1.386 (4) *vs* 1.382 (5) Å; Jourdain *et al.*, 2013 ▸]. The formation of the thermodynamic isomer, already evidenced by ^1^H NMR spectroscopy, is indicated by the attachment of the aromatic C_6_H_2_Me_3_ ring at the C2 atom in the β position relative to platinum. The C(=O)C(C_6_H_2_Me_3_)=C(H) moiety is σ-bonded to the platinum atom [C1—Pt1 = 2.023 (3) Å], which adopts an irregular shape; see Table 1 ▸. The τ_4_ descriptor for four-coord­ination is 0.39 (τ_4_ = 0 for a perfect square-planar geometry and = 1 for a perfect tetra­hedral geometry; for inter­mediate structures, including trigonal–pyramidal and seesaw, τ_4_ falls within the range 0 to 1; Yang *et al.*, 2007 ▸).

## Supra­molecular features   

**Figure 3:**
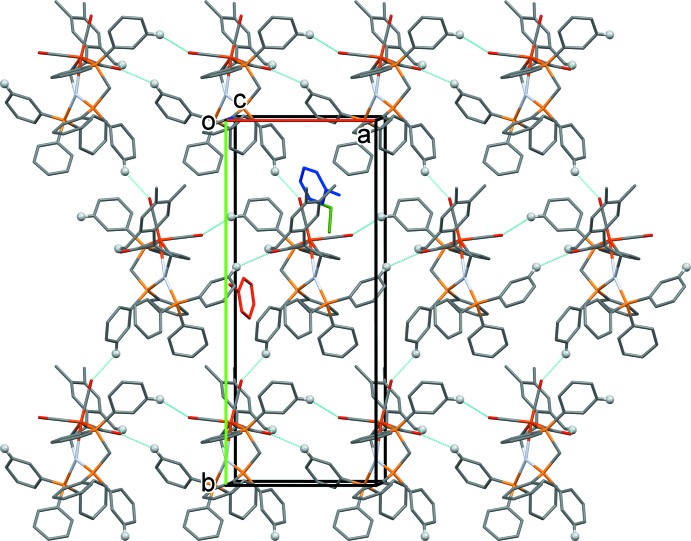
A partial view along the *c* axis of the crystal packing of the title compound. The C—H⋯O hydrogen bonds (Table 2 ▸) are shown as dashed lines. For clarity, only the H atoms (grey balls) involved in these inter­actions have been included. Colour code: the two toluene mol­ecules are red and blue and the di­chloro­methane mol­ecule is green.

In the crystal, mol­ecules are linked by a number of C—H⋯O hydrogen bonds, forming layers parallel to the *ab* plane (Fig. 3 ▸ and Table 2 ▸). There are also a number of intra- and inter­molecular C—H⋯π inter­actions present (Table 2 ▸). The methyl group involving atom C11 forms an intra­molecular C11—H11*A*⋯O1 hydrogen bond and an inter­molecular C11—H11*B*⋯π inter­action (Table 2 ▸).

## Database survey   

Other examples of crystallographically characterized dimetalla­cyclo­pentenone complexes are Fe_2_Cp_2_(CO)(μ-CO){μ-CH=C(Ph)C(=O)} (Boni *et al.*, 2011 ▸), Fe_2_Cp*_2_(CO)(μ-CO){μ-C(C≡CH)=CHC(=O)] (Akita *et al.*, 1993 ▸), Fe_2_(CO)_5_(μ-dppm){μ-C(=O)CH=CH} (Knox *et al.*, 1995 ▸), Fe_2_(CO)_5_(μ-dppm){μ-C(=O)C(Ph)=CH} (Hitchcock *et al.*, 1993 ▸), Fe_2_Cp_2_(CO)(μ-CO){μ-C(CO*R*)=C(Me)C(=O)}, where *R* = Ph, Bu (Wong *et al.*, 1991 ▸), Fe_2_{(η-C_5_H_4_)_2_SiMe_2_}(CO)_2_(μ-CO){μ-C(Ph)=C(H)C(=O)} (McKee *et al.*, 1994 ▸), Ru_2_(CO)_4_(μ-dppm)_2_{μ-C(=O)C(CO_2_Me)=C(CO_2_Me)} (Johnson & Gladfelter, 1991 ▸), Ru_2_(CO)_4_(μ-dppm)_2_{μ-CH=CHC(=O)} (Mirza *et al.*, 1994 ▸), Ru_2_(η-C_5_HMe_4_)_2_(CO)(μ-CO){μ-C(=O)C(*R*)=C(*R*)}, where *R* = Et, Me (Horiuchi *et al.*, 2012 ▸), Rh_2_Cp_2_(CO)_4_{μ-C(CF_3_)=C(CF_3_)C(=O)} (Dickson *et al.*, 1981 ▸), Re_2_Cp*_2_(CO)_2_{μ-CH=C{C(=CH_2_)CH_3_}C(=O)} (Casey *et al.*, 1994 ▸). A rare example of a heterodinuclear combination is CpFe{μ-C(=O)C(CMe_2_OH)=CH}(μ-CO)Ru(CO)Cp* (Dennett *et al.*, 2005 ▸).

## Synthesis and crystallization   

[(OC)_3_Fe(μ-CO)μ-Ph_2_PCH_2_PPh_2_)Pt(PPh_3_)] (110 mg, 0.1 mmol) was treated with an excess of 1-ethynyl-2,4,5-tri­methyl­benzene (30 mg, 0.2 mmol) in toluene (3 ml). The solution was stirred at 343 K for 2 h. The reaction mixture was filtered, and all volatiles removed under reduced pressure. The red residue was redissolved in a minimum of a di­chloro­methane/toluene mixture (50:50). Yellow crystals were isolated by layering with heptane (yield 123 mg, 88%).

Elemental analysis calculated for C_57_H_49_FeO_3_P_3_Pt, CH_2_Cl_2_, 2(C_7_H_8_) (*M*_w_ = 1395.09): C, 61.99; H, 4.84%. Found: C, 61.75; H, 4.78%. ^1^H NMR: δ 2.14 (*s*, 3H, CH_3_), 2.17 (*s*, 3H, CH_3_), 2.43 (*s*, 3H, CH_3_), 4.64 (*m*, 2H, PCH_2_P, ^2^ *J*_PH_ = 8.5, ^2^ *J*_PtH_ = 42), 6.81–7.55 (*m*, 37H, Ph), 8.07 (*dd*, 1H, =CH, ^3^ *J*_PH_ = 8.2, ^3^ *J*_PH_ = 5.0, ^2^ *J*_PtH_ = 32). ^31^P{1H} NMR: δ 8.6 (*d*, P_dppm Pt_, ^2^ *J*_PP_ = 58, ^1^ *J*_PtP_ = 2641), 33.7 (*d*, P_PPh3 Pt_, ^3^ *J*_PP_ = 36, ^1^ *J*_PtP_ = 3432), 61.3 (*dd*, P_dppm Fe_, ^2^ *J*_PP_ = 58, ^3^ *J*_PP_ = 36). IR(ATR): 1962, 1913*vs* ν(CO), 1686*m* ν(C=O).

## Refinement   

Crystal data, data collection and structure refinement details are summarized in Table 3 ▸. All of the hydrogen atoms were placed in geometrically calculated positions (C—H = 0.93–0.98 Å) and refined as riding on the parent C atom, with *U*_iso_(H) = 1.5*U*_eq_(C-meth­yl) and 1.2*U*_eq_(C) for other H atoms.

## Supplementary Material

Crystal structure: contains datablock(s) Global, I. DOI: 10.1107/S2056989019015573/su5528sup1.cif

CCDC reference: 1964051

Additional supporting information:  crystallographic information; 3D view; checkCIF report

## Figures and Tables

**Table 1:** Selected geometric parameters (Å, °)

Pt1—Fe1	2.5770 (5)	Fe1—C2	2.109 (4)
Pt1—P2	2.2700 (9)	Fe1—C12	1.929 (4)
Pt1—P3	2.2529 (9)	Fe1—C13	1.749 (4)
Pt1—C1	2.023 (3)	Fe1—C14	1.781 (4)
Fe1—P1	2.1857 (11)	O1—C12	1.207 (4)
Fe1—C1	2.107 (3)	C1—C2	1.386 (4)
			
P2—Pt1—Fe1	102.03 (3)	C1—Fe1—C2	38.38 (12)
P3—Pt1—Fe1	152.88 (3)	C13—Fe1—P1	95.17 (13)
C1—Pt1—Fe1	52.87 (10)	C14—Fe1—P1	104.63 (12)
P3—Pt1—P2	105.07 (3)	C12—Fe1—P1	94.69 (11)
C1—Pt1—P2	152.26 (10)	C1—Fe1—P1	137.46 (10)
C1—Pt1—P3	100.38 (10)	C2—Fe1—P1	135.94 (10)
Pt1—C1—Fe1	77.18 (12)	C13—Fe1—Pt1	170.69 (12)
C13—Fe1—C14	96.51 (17)	C14—Fe1—Pt1	91.16 (13)
C13—Fe1—C12	96.59 (16)	C12—Fe1—Pt1	74.38 (10)
C14—Fe1—C12	155.50 (17)	C1—Fe1—Pt1	49.95 (9)
C13—Fe1—C1	125.43 (16)	C2—Fe1—Pt1	74.35 (9)
C14—Fe1—C1	84.72 (15)	P1—Fe1—Pt1	87.95 (3)
C12—Fe1—C1	70.81 (14)	C2—C1—Pt1	112.5 (3)
C13—Fe1—C2	97.50 (15)	C2—C1—Fe1	70.9 (2)
C14—Fe1—C2	115.51 (16)	P1—C45—P2	108.83 (17)
C12—Fe1—C2	42.03 (13)		

**Table 2:** Hydrogen-bond geometry (Å, °) *Cg*3, *Cg*6, *Cg*8, *Cg*9 and *Cg*10 are the centroids of the C21–C26, C39–C44, C52–C57, C59–C64 and C66–C71 rings, respectively.

*D*—H⋯*A*	*D*—H	H⋯*A*	*D*⋯*A*	*D*—H⋯*A*
C11—H11*A*⋯O1	0.96	2.36	3.193 (4)	145
C31—H31⋯O1^i^	0.93	2.55	3.370 (5)	147
C41—H41⋯O2^ii^	0.93	2.49	3.202 (5)	134
C48—H48⋯O3^iii^	0.93	2.46	3.325 (5)	154
C11—H11*B*⋯*Cg*9^iv^	0.96	2.80	3.719 (4)	160
C22—H22⋯*Cg*6	0.93	2.80	3.597 (3)	145
C34—H34⋯*Cg*3	0.93	2.98	3.519 (4)	118
C38—H38⋯*Cg*10	0.93	2.82	3.694 (4)	156
C60—H60⋯*Cg*8	0.93	2.81	3.543 (5)	137

Symmetry codes: (i) 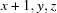; (ii) 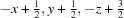; (iii) 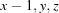; (iv) 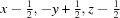.

**Table 3:** Experimental details

Crystal data	
Chemical formula	[FePt(C_12_H_12_O)(C_18_H_15_P)(C_25_H_22_P_2_)(CO)_2_]·2C_7_H_8_·CH_2_Cl_2_
*M*_r_	1395.00
Crystal system, space group	Monoclinic, *P*2_1_/*n*
Temperature (K)	293
*a*, *b*, *c* (Å)	10.2117 (3), 24.7895 (6), 24.6241 (7)
β (°)	92.056 (3)
*V* (Å^3^)	6229.4 (3)
*Z*	4
Radiation type	Mo *K*α
μ (mm^−1^)	2.69
Crystal size (mm)	0.49 × 0.39 × 0.15

Data collection	
Diffractometer	Oxford Diffraction Xcalibur, Sapphire3
Absorption correction	Multi-scan (*CrysAlis PRO*; Oxford Diffraction, 2010 ▸)
*T*_min_, *T*_max_	0.923, 1.000
No. of measured, independent and observed [*I* > 2σ(*I*)] reflections	52106, 14857, 8982
*R*_int_	0.070
(sin θ/λ)_max_ (Å^−1^)	0.687

Refinement	
*R*[*F* ^2^ > 2σ(*F* ^2^)], *wR*(*F* ^2^), *S*	0.035, 0.054, 0.81
No. of reflections	14857
No. of parameters	744
H-atom treatment	H-atom parameters constrained
Δρ_max_, Δρ_min_ (e Å^−3^)	1.52, −0.97

Computer programs: *CrysAlis PRO* (Oxford Diffraction, 2010 ▸), *SHELXT* (Sheldrick, 2015*a* ▸), *SHELXL* (Sheldrick, 2015*b* ▸), *OLEX2* (Dolomanov *et al.*, 2009 ▸), *Mercury* (Macrae *et al.*, 2008 ▸), *PLATON* (Spek, 2009 ▸) and *publCIF* (Westrip, 2010 ▸).

## supplementary crystallographic information

### Crystal data

**Table:**

[FePt(C_12_H_12_O)(C_18_H_15_P)(C_25_H_22_P_2_)(CO)_2_]·2C_7_H_8_·CH_2_Cl_2_	*F*(000) = 2824
*M**_r_* = 1395.00	*D*_x_ = 1.487 Mg m^−^^3^
Monoclinic, *P*2_1_/*n*	Mo *K*α radiation, λ = 0.71073 Å
*a* = 10.2117 (3) Å	Cell parameters from 14906 reflections
*b* = 24.7895 (6) Å	θ = 2.1–29.2°
*c* = 24.6241 (7) Å	µ = 2.69 mm^−^^1^
β = 92.056 (3)°	*T* = 293 K
*V* = 6229.4 (3) Å^3^	Block, yellow
*Z* = 4	0.49 × 0.39 × 0.14 mm

### Data collection

**Table:**

Oxford Diffraction Xcalibur, Sapphire3 diffractometer	14857 independent reflections
Radiation source: microfocus sealed X-ray tube	8982 reflections with *I* > 2σ(*I*)
Grahpite monochromator	*R*_int_ = 0.070
Detector resolution: 16.0560 pixels mm^-1^	θ_max_ = 29.2°, θ_min_ = 2.1°
ω and φ scans	*h* = −13→13
Absorption correction: multi-scan (CrysAlisPro; Oxford Diffraction, 2010)	*k* = −33→33
*T*_min_ = 0.923, *T*_max_ = 1.000	*l* = −33→33
52106 measured reflections	

### Refinement

**Table:**

Refinement on *F*^2^	Primary atom site location: dual
Least-squares matrix: full	Secondary atom site location: difference Fourier map
*R*[*F*^2^ > 2σ(*F*^2^)] = 0.035	Hydrogen site location: mixed
*wR*(*F*^2^) = 0.054	H-atom parameters constrained
*S* = 0.81	*w* = 1/[σ^2^(*F*_o_^2^) + (0.0167*P*)^2^] where *P* = (*F*_o_^2^ + 2*F*_c_^2^)/3
14857 reflections	(Δ/σ)_max_ = 0.004
744 parameters	Δρ_max_ = 1.52 e Å^−^^3^
0 restraints	Δρ_min_ = −0.97 e Å^−^^3^

### Special details

**Table:**

Geometry. All esds (except the esd in the dihedral angle between two l.s. planes) are estimated using the full covariance matrix. The cell esds are taken into account individually in the estimation of esds in distances, angles and torsion angles; correlations between esds in cell parameters are only used when they are defined by crystal symmetry. An approximate (isotropic) treatment of cell esds is used for estimating esds involving l.s. planes.

### Fractional atomic coordinates and isotropic or equivalent isotropic displacement parameters (Å^2^)

**Table:**

	*x*	*y*	*z*	*U*_iso_*/*U*_eq_	
Pt1	0.54891 (2)	0.43749 (2)	0.70894 (2)	0.01562 (4)	
Fe1	0.51432 (5)	0.33557 (2)	0.72321 (2)	0.01800 (12)	
P1	0.41192 (9)	0.35546 (4)	0.79703 (4)	0.0181 (2)	
P2	0.42967 (9)	0.47366 (4)	0.77594 (4)	0.0166 (2)	
P3	0.63044 (9)	0.50877 (4)	0.66456 (4)	0.0174 (2)	
O1	0.2571 (2)	0.36786 (10)	0.67002 (10)	0.0277 (7)	
O2	0.4463 (3)	0.22195 (10)	0.72360 (12)	0.0468 (8)	
O3	0.7908 (3)	0.32228 (13)	0.75629 (11)	0.0529 (9)	
C1	0.5924 (3)	0.37724 (14)	0.65717 (13)	0.0202 (9)	
H1	0.680375	0.370787	0.643864	0.024*	
C2	0.4823 (3)	0.34885 (14)	0.63921 (15)	0.0207 (9)	
C3	0.4833 (3)	0.30712 (15)	0.59518 (14)	0.0211 (9)	
C4	0.5756 (4)	0.26604 (15)	0.59748 (15)	0.0278 (10)	
H4	0.632062	0.264056	0.627904	0.033*	
C5	0.5883 (4)	0.22799 (16)	0.55717 (16)	0.0317 (10)	
C6	0.6912 (4)	0.18437 (17)	0.56150 (17)	0.0565 (14)	
H6A	0.747993	0.190977	0.592699	0.085*	
H6B	0.741631	0.184430	0.529337	0.085*	
H6C	0.649419	0.149939	0.565208	0.085*	
C7	0.5028 (4)	0.23059 (15)	0.51200 (16)	0.0323 (10)	
C8	0.5085 (4)	0.19051 (17)	0.46646 (16)	0.0475 (12)	
H8A	0.500249	0.154669	0.480700	0.071*	
H8B	0.590835	0.193877	0.449098	0.071*	
H8C	0.438224	0.197341	0.440447	0.071*	
C9	0.4092 (4)	0.27137 (16)	0.50997 (15)	0.0304 (10)	
H9	0.351454	0.272838	0.479938	0.036*	
C10	0.3974 (3)	0.30992 (15)	0.55019 (14)	0.0240 (9)	
C11	0.2989 (4)	0.35501 (16)	0.54280 (15)	0.0372 (11)	
H11A	0.248433	0.358014	0.574796	0.056*	
H11B	0.241625	0.347360	0.512004	0.056*	
H11C	0.344170	0.388334	0.536886	0.056*	
C12	0.3729 (4)	0.35839 (14)	0.67444 (14)	0.0207 (9)	
C13	0.4732 (4)	0.26710 (16)	0.72377 (15)	0.0272 (9)	
C14	0.6811 (4)	0.32730 (16)	0.74531 (15)	0.0289 (10)	
C15	0.5260 (3)	0.53319 (15)	0.60843 (14)	0.0185 (8)	
C16	0.4393 (3)	0.49690 (15)	0.58390 (14)	0.0241 (9)	
H16	0.435154	0.461613	0.596549	0.029*	
C17	0.3586 (4)	0.51324 (17)	0.54045 (15)	0.0319 (10)	
H17	0.301231	0.488704	0.523745	0.038*	
C18	0.3630 (4)	0.56526 (19)	0.52205 (15)	0.0367 (10)	
H18	0.309049	0.575926	0.492759	0.044*	
C19	0.4470 (4)	0.60178 (16)	0.54680 (16)	0.0357 (11)	
H19	0.448309	0.637397	0.534981	0.043*	
C20	0.5286 (4)	0.58554 (16)	0.58877 (15)	0.0282 (10)	
H20	0.587186	0.610160	0.604539	0.034*	
C21	0.6723 (3)	0.56935 (14)	0.70350 (13)	0.0184 (8)	
C22	0.5742 (3)	0.60459 (14)	0.71862 (14)	0.0228 (9)	
H22	0.488474	0.599401	0.705593	0.027*	
C23	0.6035 (4)	0.64710 (15)	0.75278 (15)	0.0285 (10)	
H23	0.537028	0.670266	0.762908	0.034*	
C24	0.7283 (4)	0.65583 (15)	0.77201 (15)	0.0300 (10)	
H24	0.747113	0.684953	0.794782	0.036*	
C25	0.8256 (4)	0.62155 (15)	0.75764 (15)	0.0295 (10)	
H25	0.910855	0.627190	0.771023	0.035*	
C26	0.7987 (4)	0.57856 (14)	0.72340 (14)	0.0246 (9)	
H26	0.866047	0.555636	0.713645	0.030*	
C27	0.7855 (3)	0.49061 (14)	0.63509 (15)	0.0220 (9)	
C28	0.8308 (4)	0.51595 (17)	0.58964 (15)	0.0350 (11)	
H28	0.782037	0.543429	0.573041	0.042*	
C29	0.9485 (5)	0.5005 (2)	0.56880 (19)	0.0564 (15)	
H29	0.978621	0.517932	0.538205	0.068*	
C30	1.0213 (5)	0.4604 (2)	0.5922 (2)	0.0582 (16)	
H30	1.099617	0.449824	0.577150	0.070*	
C31	0.9788 (4)	0.43555 (18)	0.63789 (19)	0.0445 (12)	
H31	1.029448	0.408646	0.654527	0.053*	
C32	0.8601 (3)	0.45036 (14)	0.65964 (16)	0.0306 (10)	
H32	0.831182	0.433221	0.690593	0.037*	
C33	0.5029 (3)	0.49956 (13)	0.83862 (14)	0.0163 (8)	
C34	0.6295 (4)	0.51743 (14)	0.83861 (15)	0.0240 (9)	
H34	0.676634	0.514638	0.807096	0.029*	
C35	0.6883 (4)	0.53955 (16)	0.88470 (16)	0.0315 (10)	
H35	0.774147	0.552029	0.884128	0.038*	
C36	0.6194 (4)	0.54305 (16)	0.93152 (16)	0.0342 (11)	
H36	0.658776	0.557529	0.962884	0.041*	
C37	0.4921 (4)	0.52508 (16)	0.93185 (14)	0.0294 (10)	
H37	0.444711	0.527762	0.963299	0.035*	
C38	0.4356 (3)	0.50336 (14)	0.88599 (14)	0.0223 (9)	
H38	0.349785	0.490820	0.886621	0.027*	
C39	0.3165 (3)	0.52640 (13)	0.75401 (14)	0.0157 (8)	
C40	0.3041 (3)	0.57485 (13)	0.78128 (15)	0.0250 (9)	
H40	0.349673	0.580082	0.814244	0.030*	
C41	0.2253 (4)	0.61542 (15)	0.76031 (16)	0.0316 (10)	
H41	0.218062	0.647762	0.779109	0.038*	
C42	0.1575 (4)	0.60827 (15)	0.71186 (16)	0.0298 (10)	
H42	0.104378	0.635668	0.697618	0.036*	
C43	0.1685 (3)	0.56021 (17)	0.68438 (15)	0.0319 (9)	
H43	0.122081	0.555114	0.651600	0.038*	
C44	0.2475 (3)	0.51976 (14)	0.70495 (14)	0.0232 (9)	
H44	0.254809	0.487621	0.685819	0.028*	
C45	0.3186 (3)	0.41845 (12)	0.79317 (13)	0.0167 (8)	
H45A	0.279720	0.425680	0.827798	0.020*	
H43B	0.248605	0.415473	0.765633	0.020*	
C46	0.2875 (3)	0.30636 (14)	0.81481 (13)	0.0171 (8)	
C47	0.1619 (4)	0.30890 (15)	0.79365 (15)	0.0307 (10)	
H47	0.137670	0.337375	0.770842	0.037*	
C48	0.0702 (4)	0.26986 (17)	0.80557 (17)	0.0406 (12)	
H48	−0.014739	0.272452	0.790848	0.049*	
C49	0.1037 (4)	0.22774 (15)	0.83871 (16)	0.0323 (10)	
H49	0.041725	0.201813	0.847043	0.039*	
C50	0.2290 (4)	0.22384 (15)	0.85963 (15)	0.0310 (10)	
H50	0.253127	0.195127	0.882155	0.037*	
C51	0.3195 (4)	0.26267 (15)	0.84714 (15)	0.0294 (10)	
H51	0.404964	0.259288	0.861023	0.035*	
C52	0.5016 (3)	0.36484 (14)	0.86208 (14)	0.0198 (9)	
C53	0.6322 (4)	0.37850 (14)	0.86408 (15)	0.0255 (9)	
H53	0.677321	0.381007	0.832040	0.031*	
C54	0.6971 (4)	0.38856 (16)	0.91340 (16)	0.0318 (10)	
H54	0.785234	0.398083	0.914070	0.038*	
C55	0.6334 (4)	0.38468 (16)	0.96099 (16)	0.0348 (11)	
H55	0.678280	0.390548	0.994001	0.042*	
C56	0.5024 (4)	0.37203 (15)	0.95967 (15)	0.0340 (11)	
H56	0.458020	0.369732	0.991924	0.041*	
C57	0.4365 (4)	0.36275 (14)	0.91088 (15)	0.0251 (9)	
H57	0.347377	0.354967	0.910412	0.030*	
C58	0.7015 (5)	0.21333 (19)	0.86522 (18)	0.0629 (15)	
H58A	0.767069	0.185760	0.862975	0.094*	
H58B	0.742922	0.247326	0.873172	0.094*	
H58C	0.652954	0.215744	0.831189	0.094*	
C59	0.6110 (5)	0.1997 (2)	0.90902 (19)	0.0455 (12)	
C60	0.5850 (5)	0.23732 (19)	0.9492 (2)	0.0520 (13)	
H60	0.626737	0.270661	0.948931	0.062*	
C61	0.4975 (5)	0.2259 (2)	0.9899 (2)	0.0636 (16)	
H61	0.482133	0.251653	1.016462	0.076*	
C62	0.4343 (5)	0.1775 (2)	0.9912 (2)	0.0716 (17)	
H62	0.374855	0.169870	1.017964	0.086*	
C63	0.4614 (5)	0.1401 (2)	0.9516 (2)	0.0700 (17)	
H63	0.420309	0.106633	0.951855	0.084*	
C64	0.5489 (5)	0.1516 (2)	0.9113 (2)	0.0532 (14)	
H64	0.565255	0.125629	0.885175	0.064*	
C65	−0.0246 (4)	0.4584 (2)	0.8046 (2)	0.085 (2)	
H65A	−0.025429	0.419702	0.804299	0.128*	
H65B	0.028702	0.471343	0.776000	0.128*	
H65C	−0.112369	0.471694	0.799097	0.128*	
C66	0.0300 (4)	0.4778 (2)	0.8576 (2)	0.0549 (14)	
C67	0.0512 (5)	0.5325 (3)	0.8671 (3)	0.085 (2)	
H67	0.032387	0.557028	0.839253	0.102*	
C68	0.0994 (6)	0.5514 (3)	0.9166 (4)	0.099 (3)	
H68	0.112688	0.588053	0.922887	0.119*	
C69	0.1268 (5)	0.5136 (3)	0.9564 (3)	0.082 (2)	
H69	0.157815	0.525130	0.990425	0.098*	
C70	0.1098 (5)	0.4592 (3)	0.9473 (2)	0.0660 (16)	
H70	0.131156	0.434384	0.974479	0.079*	
C71	0.0617 (4)	0.4422 (2)	0.89843 (19)	0.0512 (13)	
H71	0.049822	0.405504	0.892407	0.061*	
Cl1	0.61998 (19)	0.31739 (7)	1.11202 (6)	0.1133 (6)	
Cl2	0.47570 (16)	0.22636 (7)	1.14958 (6)	0.0936 (5)	
C72	0.6305 (5)	0.2515 (2)	1.1363 (2)	0.0799 (17)	
H72A	0.685110	0.250711	1.169320	0.096*	
H72B	0.671131	0.228837	1.109553	0.096*	

### Atomic displacement parameters (Å^2^)

**Table:**

	*U*^11^	*U*^22^	*U*^33^	*U*^12^	*U*^13^	*U*^23^
Pt1	0.01643 (7)	0.01350 (6)	0.01695 (7)	0.00082 (8)	0.00098 (5)	−0.00172 (8)
Fe1	0.0182 (3)	0.0137 (3)	0.0221 (3)	0.0020 (2)	0.0000 (2)	−0.0001 (2)
P1	0.0171 (5)	0.0158 (5)	0.0214 (6)	−0.0004 (4)	−0.0008 (5)	0.0012 (4)
P2	0.0173 (5)	0.0152 (5)	0.0174 (5)	0.0013 (4)	0.0009 (4)	−0.0023 (4)
P3	0.0183 (5)	0.0155 (5)	0.0184 (5)	0.0000 (4)	0.0018 (4)	−0.0015 (4)
O1	0.0141 (15)	0.0360 (17)	0.0330 (17)	0.0090 (12)	−0.0007 (13)	−0.0043 (13)
O2	0.062 (2)	0.0146 (15)	0.064 (2)	−0.0073 (15)	0.0116 (17)	−0.0017 (15)
O3	0.0243 (18)	0.080 (3)	0.054 (2)	0.0202 (17)	−0.0047 (17)	−0.0024 (18)
C1	0.016 (2)	0.023 (2)	0.021 (2)	0.0030 (16)	−0.0019 (17)	0.0014 (17)
C2	0.023 (2)	0.0132 (19)	0.026 (2)	0.0020 (16)	0.0043 (18)	−0.0015 (17)
C3	0.022 (2)	0.020 (2)	0.021 (2)	−0.0015 (17)	0.0039 (18)	−0.0013 (18)
C4	0.030 (2)	0.028 (2)	0.025 (2)	0.0030 (19)	−0.0074 (19)	−0.009 (2)
C5	0.037 (3)	0.028 (2)	0.030 (2)	0.009 (2)	0.002 (2)	−0.012 (2)
C6	0.069 (4)	0.047 (3)	0.052 (3)	0.028 (3)	−0.014 (3)	−0.024 (3)
C7	0.045 (3)	0.023 (2)	0.029 (3)	0.000 (2)	0.001 (2)	−0.010 (2)
C8	0.062 (3)	0.038 (3)	0.042 (3)	0.004 (2)	−0.001 (2)	−0.015 (2)
C9	0.036 (3)	0.032 (2)	0.022 (2)	−0.003 (2)	−0.007 (2)	−0.003 (2)
C10	0.024 (2)	0.026 (2)	0.021 (2)	−0.0031 (18)	−0.0033 (18)	−0.0031 (19)
C11	0.040 (3)	0.037 (3)	0.034 (3)	0.006 (2)	−0.010 (2)	−0.003 (2)
C12	0.025 (2)	0.016 (2)	0.022 (2)	−0.0022 (17)	0.0021 (19)	0.0028 (17)
C13	0.025 (2)	0.027 (2)	0.030 (2)	0.0008 (19)	0.0043 (19)	0.002 (2)
C14	0.032 (3)	0.029 (2)	0.025 (2)	0.004 (2)	0.000 (2)	−0.0013 (19)
C15	0.020 (2)	0.020 (2)	0.016 (2)	0.0008 (17)	0.0064 (17)	0.0031 (17)
C16	0.024 (2)	0.026 (2)	0.022 (2)	0.0020 (18)	−0.0017 (18)	0.0048 (19)
C17	0.030 (3)	0.039 (3)	0.026 (2)	−0.009 (2)	−0.007 (2)	0.000 (2)
C18	0.038 (3)	0.050 (3)	0.021 (2)	0.014 (3)	−0.0043 (19)	0.008 (3)
C19	0.048 (3)	0.027 (2)	0.032 (3)	0.002 (2)	−0.002 (2)	0.009 (2)
C20	0.036 (3)	0.024 (2)	0.024 (2)	−0.0014 (19)	−0.003 (2)	0.0008 (19)
C21	0.023 (2)	0.0134 (19)	0.0191 (19)	−0.0069 (17)	0.0025 (16)	0.0014 (17)
C22	0.023 (2)	0.020 (2)	0.025 (2)	−0.0041 (17)	0.0030 (18)	0.0003 (18)
C23	0.034 (3)	0.023 (2)	0.029 (2)	0.0077 (19)	0.011 (2)	−0.0020 (19)
C24	0.041 (3)	0.020 (2)	0.029 (2)	−0.008 (2)	0.002 (2)	−0.0083 (19)
C25	0.027 (2)	0.030 (2)	0.030 (2)	−0.0094 (19)	−0.006 (2)	0.001 (2)
C26	0.028 (2)	0.019 (2)	0.027 (2)	0.0013 (16)	0.0004 (19)	−0.0024 (17)
C27	0.022 (2)	0.021 (2)	0.023 (2)	−0.0045 (17)	−0.0017 (18)	−0.0068 (18)
C28	0.039 (3)	0.044 (3)	0.023 (2)	−0.004 (2)	0.003 (2)	−0.009 (2)
C29	0.046 (3)	0.088 (4)	0.037 (3)	−0.020 (3)	0.017 (3)	−0.027 (3)
C30	0.031 (3)	0.070 (4)	0.075 (4)	−0.001 (3)	0.019 (3)	−0.044 (3)
C31	0.019 (2)	0.032 (2)	0.083 (4)	0.002 (2)	0.000 (2)	−0.019 (3)
C32	0.020 (2)	0.023 (2)	0.049 (3)	−0.0016 (17)	0.002 (2)	−0.003 (2)
C33	0.020 (2)	0.0106 (18)	0.018 (2)	0.0033 (16)	−0.0008 (17)	−0.0029 (16)
C34	0.025 (2)	0.026 (2)	0.022 (2)	0.0025 (18)	0.0084 (19)	0.0006 (19)
C35	0.023 (2)	0.038 (3)	0.034 (3)	−0.0082 (19)	−0.004 (2)	−0.007 (2)
C36	0.039 (3)	0.040 (3)	0.023 (2)	−0.004 (2)	−0.005 (2)	−0.007 (2)
C37	0.036 (3)	0.039 (3)	0.013 (2)	0.001 (2)	0.0035 (19)	−0.002 (2)
C38	0.023 (2)	0.023 (2)	0.022 (2)	−0.0012 (17)	−0.0012 (18)	−0.0043 (18)
C39	0.015 (2)	0.0116 (18)	0.020 (2)	0.0006 (15)	0.0014 (17)	0.0026 (16)
C40	0.029 (2)	0.019 (2)	0.026 (2)	0.0088 (17)	−0.0024 (18)	−0.0027 (17)
C41	0.033 (3)	0.021 (2)	0.040 (3)	0.0089 (19)	0.001 (2)	−0.008 (2)
C42	0.028 (2)	0.025 (2)	0.036 (3)	0.0117 (19)	−0.003 (2)	0.010 (2)
C43	0.031 (2)	0.033 (2)	0.031 (2)	0.002 (2)	−0.0112 (18)	−0.005 (2)
C44	0.027 (2)	0.019 (2)	0.025 (2)	0.0036 (18)	0.0025 (19)	−0.0036 (18)
C45	0.018 (2)	0.0178 (19)	0.0144 (19)	0.0003 (15)	−0.0032 (16)	−0.0026 (16)
C46	0.016 (2)	0.018 (2)	0.017 (2)	0.0009 (16)	−0.0001 (17)	−0.0047 (17)
C47	0.029 (2)	0.023 (2)	0.040 (3)	−0.0050 (19)	−0.005 (2)	0.012 (2)
C48	0.021 (2)	0.039 (3)	0.060 (3)	−0.003 (2)	−0.013 (2)	0.012 (2)
C49	0.030 (3)	0.024 (2)	0.043 (3)	−0.0107 (19)	0.006 (2)	0.003 (2)
C50	0.034 (3)	0.023 (2)	0.035 (3)	−0.0045 (19)	−0.007 (2)	0.011 (2)
C51	0.019 (2)	0.030 (2)	0.039 (3)	0.0005 (18)	−0.0033 (19)	0.002 (2)
C52	0.020 (2)	0.017 (2)	0.022 (2)	−0.0002 (16)	−0.0040 (18)	0.0018 (17)
C53	0.024 (2)	0.026 (2)	0.026 (2)	−0.0038 (18)	−0.0051 (19)	−0.0004 (19)
C54	0.026 (2)	0.031 (2)	0.038 (3)	−0.0014 (19)	−0.010 (2)	−0.002 (2)
C55	0.045 (3)	0.033 (2)	0.025 (3)	−0.003 (2)	−0.013 (2)	−0.003 (2)
C56	0.049 (3)	0.034 (3)	0.019 (2)	0.000 (2)	0.004 (2)	−0.004 (2)
C57	0.024 (2)	0.027 (2)	0.024 (2)	−0.0006 (18)	−0.0008 (19)	−0.0027 (19)
C58	0.071 (4)	0.061 (4)	0.057 (3)	0.012 (3)	−0.002 (3)	0.008 (3)
C59	0.051 (3)	0.041 (3)	0.043 (3)	0.010 (3)	−0.017 (3)	0.000 (3)
C60	0.057 (3)	0.036 (3)	0.062 (4)	0.006 (3)	−0.021 (3)	−0.003 (3)
C61	0.071 (4)	0.059 (4)	0.060 (4)	0.023 (3)	−0.017 (3)	−0.015 (3)
C62	0.067 (4)	0.061 (4)	0.085 (5)	−0.003 (3)	−0.018 (3)	0.001 (4)
C63	0.067 (4)	0.053 (4)	0.088 (5)	−0.010 (3)	−0.023 (4)	−0.006 (4)
C64	0.060 (4)	0.040 (3)	0.058 (4)	−0.002 (3)	−0.018 (3)	−0.010 (3)
C65	0.037 (3)	0.151 (6)	0.069 (4)	0.026 (3)	0.001 (3)	0.019 (4)
C66	0.022 (3)	0.076 (4)	0.068 (4)	0.016 (3)	0.017 (3)	0.025 (3)
C67	0.034 (4)	0.075 (5)	0.148 (7)	0.020 (3)	0.036 (4)	0.042 (5)
C68	0.042 (4)	0.087 (6)	0.172 (8)	0.003 (4)	0.045 (5)	0.000 (6)
C69	0.049 (4)	0.103 (6)	0.096 (5)	−0.021 (4)	0.043 (4)	−0.041 (5)
C70	0.053 (4)	0.086 (5)	0.060 (4)	−0.008 (3)	0.022 (3)	0.007 (3)
C71	0.037 (3)	0.067 (4)	0.050 (3)	0.000 (3)	0.011 (2)	0.019 (3)
Cl1	0.1933 (19)	0.0721 (12)	0.0726 (11)	−0.0196 (12)	−0.0191 (12)	−0.0096 (9)
Cl2	0.0980 (13)	0.0890 (13)	0.0943 (12)	0.0133 (10)	0.0097 (10)	0.0026 (10)
C72	0.083 (4)	0.055 (4)	0.101 (5)	0.008 (3)	−0.002 (4)	−0.002 (3)

### Geometric parameters (Å, º)

**Table:**

Pt1—Fe1	2.5770 (5)	C33—C38	1.379 (4)
Pt1—P2	2.2700 (9)	C34—C35	1.378 (5)
Pt1—P3	2.2529 (9)	C34—H34	0.9300
Pt1—C1	2.023 (3)	C35—C36	1.375 (5)
Fe1—P1	2.1857 (11)	C35—H35	0.9300
Fe1—C1	2.107 (3)	C36—C37	1.375 (5)
Fe1—C2	2.109 (4)	C36—H36	0.9300
Fe1—C12	1.929 (4)	C37—C38	1.360 (5)
Fe1—C13	1.749 (4)	C37—H37	0.9300
Fe1—C14	1.781 (4)	C38—H38	0.9300
P1—C46	1.824 (4)	C39—C40	1.384 (4)
P1—C45	1.830 (3)	C39—C44	1.386 (4)
P1—C52	1.831 (3)	C40—C41	1.377 (5)
P2—C33	1.809 (3)	C40—H40	0.9300
P2—C39	1.814 (3)	C41—C42	1.369 (5)
P2—C45	1.837 (3)	C41—H41	0.9300
P3—C15	1.819 (4)	C42—C43	1.377 (5)
P3—C27	1.822 (4)	C42—H42	0.9300
P3—C21	1.824 (3)	C43—C44	1.372 (5)
O1—C12	1.207 (4)	C43—H43	0.9300
O2—C13	1.152 (4)	C44—H44	0.9300
O3—C14	1.149 (4)	C45—H45A	0.9700
C1—C2	1.386 (4)	C45—H43B	0.9700
C1—H1	0.9806	C46—C47	1.369 (4)
C2—C12	1.458 (5)	C46—C51	1.376 (5)
C2—C3	1.499 (5)	C47—C48	1.386 (5)
C3—C4	1.387 (5)	C47—H47	0.9300
C3—C10	1.390 (4)	C48—C49	1.362 (5)
C4—C5	1.379 (5)	C48—H48	0.9300
C4—H4	0.9300	C49—C50	1.366 (5)
C5—C7	1.391 (5)	C49—H49	0.9300
C5—C6	1.509 (5)	C50—C51	1.376 (5)
C6—H6A	0.9600	C50—H50	0.9300
C6—H6B	0.9600	C51—H51	0.9300
C6—H6C	0.9600	C52—C53	1.375 (4)
C7—C9	1.391 (5)	C52—C57	1.395 (5)
C7—C8	1.501 (5)	C53—C54	1.385 (5)
C8—H8A	0.9600	C53—H53	0.9300
C8—H8B	0.9600	C54—C55	1.364 (5)
C8—H8C	0.9600	C54—H54	0.9300
C9—C10	1.385 (5)	C55—C56	1.374 (5)
C9—H9	0.9300	C55—H55	0.9300
C10—C11	1.510 (5)	C56—C57	1.375 (5)
C11—H11A	0.9600	C56—H56	0.9300
C11—H11B	0.9600	C57—H57	0.9300
C11—H11C	0.9600	C58—C59	1.484 (6)
C15—C16	1.386 (5)	C58—H58A	0.9600
C15—C20	1.386 (5)	C58—H58B	0.9600
C16—C17	1.387 (5)	C58—H58C	0.9600
C16—H16	0.9300	C59—C64	1.351 (6)
C17—C18	1.368 (5)	C59—C60	1.393 (6)
C17—H17	0.9300	C60—C61	1.395 (6)
C18—C19	1.375 (5)	C60—H60	0.9300
C18—H18	0.9300	C61—C62	1.365 (7)
C19—C20	1.364 (5)	C61—H61	0.9300
C19—H19	0.9300	C62—C63	1.382 (7)
C20—H20	0.9300	C62—H62	0.9300
C21—C26	1.383 (4)	C63—C64	1.389 (6)
C21—C22	1.390 (4)	C63—H63	0.9300
C22—C23	1.375 (5)	C64—H64	0.9300
C22—H22	0.9300	C65—C66	1.481 (6)
C23—C24	1.361 (5)	C65—H65A	0.9600
C23—H23	0.9300	C65—H65B	0.9600
C24—C25	1.364 (5)	C65—H65C	0.9600
C24—H24	0.9300	C66—C71	1.366 (6)
C25—C26	1.380 (5)	C66—C67	1.393 (7)
C25—H25	0.9300	C67—C68	1.382 (8)
C26—H26	0.9300	C67—H67	0.9300
C27—C28	1.378 (5)	C68—C69	1.377 (8)
C27—C32	1.381 (5)	C68—H68	0.9300
C28—C29	1.377 (6)	C69—C70	1.377 (7)
C28—H28	0.9300	C69—H69	0.9300
C29—C30	1.358 (6)	C70—C71	1.349 (6)
C29—H29	0.9300	C70—H70	0.9300
C30—C31	1.368 (6)	C71—H71	0.9300
C30—H30	0.9300	Cl1—C72	1.741 (5)
C31—C32	1.392 (5)	Cl2—C72	1.741 (5)
C31—H31	0.9300	C72—H72A	0.9700
C32—H32	0.9300	C72—H72B	0.9700
C33—C34	1.367 (4)		
			
P2—Pt1—Fe1	102.03 (3)	C28—C29—H29	119.4
P3—Pt1—Fe1	152.88 (3)	C29—C30—C31	119.6 (4)
C1—Pt1—Fe1	52.87 (10)	C29—C30—H30	120.2
P3—Pt1—P2	105.07 (3)	C31—C30—H30	120.2
C1—Pt1—P2	152.26 (10)	C30—C31—C32	120.3 (4)
C1—Pt1—P3	100.38 (10)	C30—C31—H31	119.8
Pt1—C1—Fe1	77.18 (12)	C32—C31—H31	119.8
C13—Fe1—C14	96.51 (17)	C27—C32—C31	119.7 (4)
C13—Fe1—C12	96.59 (16)	C27—C32—H32	120.1
C14—Fe1—C12	155.50 (17)	C31—C32—H32	120.1
C13—Fe1—C1	125.43 (16)	C34—C33—C38	118.6 (3)
C14—Fe1—C1	84.72 (15)	C34—C33—P2	118.5 (3)
C12—Fe1—C1	70.81 (14)	C38—C33—P2	122.9 (3)
C13—Fe1—C2	97.50 (15)	C33—C34—C35	120.8 (3)
C14—Fe1—C2	115.51 (16)	C33—C34—H34	119.6
C12—Fe1—C2	42.03 (13)	C35—C34—H34	119.6
C1—Fe1—C2	38.38 (12)	C36—C35—C34	119.6 (4)
C13—Fe1—P1	95.17 (13)	C36—C35—H35	120.2
C14—Fe1—P1	104.63 (12)	C34—C35—H35	120.2
C12—Fe1—P1	94.69 (11)	C37—C36—C35	119.8 (4)
C1—Fe1—P1	137.46 (10)	C37—C36—H36	120.1
C2—Fe1—P1	135.94 (10)	C35—C36—H36	120.1
C13—Fe1—Pt1	170.69 (12)	C38—C37—C36	119.8 (4)
C14—Fe1—Pt1	91.16 (13)	C38—C37—H37	120.1
C12—Fe1—Pt1	74.38 (10)	C36—C37—H37	120.1
C1—Fe1—Pt1	49.95 (9)	C37—C38—C33	121.3 (3)
C2—Fe1—Pt1	74.35 (9)	C37—C38—H38	119.3
P1—Fe1—Pt1	87.95 (3)	C33—C38—H38	119.3
C2—C1—Pt1	112.5 (3)	C40—C39—C44	118.2 (3)
C2—C1—Fe1	70.9 (2)	C40—C39—P2	123.3 (3)
C46—P1—C45	102.47 (15)	C44—C39—P2	118.2 (3)
C46—P1—C52	101.87 (16)	C41—C40—C39	120.9 (3)
C45—P1—C52	100.39 (15)	C41—C40—H40	119.5
C46—P1—Fe1	114.02 (11)	C39—C40—H40	119.5
C45—P1—Fe1	114.40 (11)	C42—C41—C40	120.2 (4)
C52—P1—Fe1	121.13 (12)	C42—C41—H41	119.9
C33—P2—C39	103.75 (16)	C40—C41—H41	119.9
C33—P2—C45	107.90 (16)	C41—C42—C43	119.5 (3)
C39—P2—C45	102.34 (15)	C41—C42—H42	120.2
C33—P2—Pt1	122.98 (12)	C43—C42—H42	120.2
C39—P2—Pt1	114.88 (11)	C44—C43—C42	120.5 (3)
C45—P2—Pt1	103.02 (11)	C44—C43—H43	119.7
C15—P3—C27	105.92 (16)	C42—C43—H43	119.7
C15—P3—C21	104.21 (16)	C43—C44—C39	120.6 (3)
C27—P3—C21	102.83 (16)	C43—C44—H44	119.7
C15—P3—Pt1	114.37 (12)	C39—C44—H44	119.7
C27—P3—Pt1	109.93 (12)	P1—C45—P2	108.83 (17)
C21—P3—Pt1	118.32 (11)	P1—C45—H45A	109.9
C2—C1—H1	123.6	P2—C45—H45A	109.9
Pt1—C1—H1	123.7	P1—C45—H43B	109.9
Fe1—C1—H1	123.7	P2—C45—H43B	109.9
C1—C2—C12	111.0 (3)	H45A—C45—H43B	108.3
C1—C2—C3	123.7 (3)	C47—C46—C51	117.1 (3)
C12—C2—C3	124.6 (3)	C47—C46—P1	122.0 (3)
C1—C2—Fe1	70.8 (2)	C51—C46—P1	120.8 (3)
C12—C2—Fe1	62.4 (2)	C46—C47—C48	121.2 (4)
C3—C2—Fe1	126.7 (2)	C46—C47—H47	119.4
C4—C3—C10	118.5 (3)	C48—C47—H47	119.4
C4—C3—C2	120.0 (3)	C49—C48—C47	120.4 (4)
C10—C3—C2	121.4 (3)	C49—C48—H48	119.8
C5—C4—C3	123.6 (3)	C47—C48—H48	119.8
C5—C4—H4	118.2	C48—C49—C50	119.5 (4)
C3—C4—H4	118.2	C48—C49—H49	120.3
C4—C5—C7	118.1 (4)	C50—C49—H49	120.3
C4—C5—C6	121.5 (3)	C49—C50—C51	119.6 (4)
C7—C5—C6	120.4 (3)	C49—C50—H50	120.2
C5—C6—H6A	109.5	C51—C50—H50	120.2
C5—C6—H6B	109.5	C46—C51—C50	122.2 (3)
H6A—C6—H6B	109.5	C46—C51—H51	118.9
C5—C6—H6C	109.5	C50—C51—H51	118.9
H6A—C6—H6C	109.5	C53—C52—C57	118.0 (3)
H6B—C6—H6C	109.5	C53—C52—P1	121.1 (3)
C5—C7—C9	118.3 (4)	C57—C52—P1	120.7 (3)
C5—C7—C8	121.8 (4)	C52—C53—C54	120.6 (4)
C9—C7—C8	120.0 (4)	C52—C53—H53	119.7
C7—C8—H8A	109.5	C54—C53—H53	119.7
C7—C8—H8B	109.5	C55—C54—C53	120.8 (4)
H8A—C8—H8B	109.5	C55—C54—H54	119.6
C7—C8—H8C	109.5	C53—C54—H54	119.6
H8A—C8—H8C	109.5	C54—C55—C56	119.3 (4)
H8B—C8—H8C	109.5	C54—C55—H55	120.3
C10—C9—C7	123.6 (4)	C56—C55—H55	120.3
C10—C9—H9	118.2	C55—C56—C57	120.3 (4)
C7—C9—H9	118.2	C55—C56—H56	119.9
C9—C10—C3	117.8 (3)	C57—C56—H56	119.9
C9—C10—C11	120.0 (3)	C56—C57—C52	120.9 (4)
C3—C10—C11	122.1 (3)	C56—C57—H57	119.6
C10—C11—H11A	109.5	C52—C57—H57	119.6
C10—C11—H11B	109.5	C59—C58—H58A	109.5
H11A—C11—H11B	109.5	C59—C58—H58B	109.5
C10—C11—H11C	109.5	H58A—C58—H58B	109.5
H11A—C11—H11C	109.5	C59—C58—H58C	109.5
H11B—C11—H11C	109.5	H58A—C58—H58C	109.5
O1—C12—C2	138.3 (3)	H58B—C58—H58C	109.5
O1—C12—Fe1	145.3 (3)	C64—C59—C60	117.4 (5)
C2—C12—Fe1	75.6 (2)	C64—C59—C58	122.4 (5)
O2—C13—Fe1	179.3 (4)	C60—C59—C58	120.2 (5)
O3—C14—Fe1	175.8 (4)	C59—C60—C61	121.2 (5)
C16—C15—C20	118.4 (3)	C59—C60—H60	119.4
C16—C15—P3	117.6 (3)	C61—C60—H60	119.4
C20—C15—P3	124.0 (3)	C62—C61—C60	120.7 (5)
C15—C16—C17	120.0 (4)	C62—C61—H61	119.6
C15—C16—H16	120.0	C60—C61—H61	119.6
C17—C16—H16	120.0	C61—C62—C63	117.8 (6)
C18—C17—C16	120.3 (4)	C61—C62—H62	121.1
C18—C17—H17	119.8	C63—C62—H62	121.1
C16—C17—H17	119.8	C62—C63—C64	121.2 (5)
C17—C18—C19	120.1 (4)	C62—C63—H63	119.4
C17—C18—H18	120.0	C64—C63—H63	119.4
C19—C18—H18	120.0	C59—C64—C63	121.7 (5)
C20—C19—C18	119.7 (4)	C59—C64—H64	119.1
C20—C19—H19	120.1	C63—C64—H64	119.1
C18—C19—H19	120.1	C66—C65—H65A	109.5
C19—C20—C15	121.5 (4)	C66—C65—H65B	109.5
C19—C20—H20	119.3	H65A—C65—H65B	109.5
C15—C20—H20	119.3	C66—C65—H65C	109.5
C26—C21—C22	118.3 (3)	H65A—C65—H65C	109.5
C26—C21—P3	121.3 (3)	H65B—C65—H65C	109.5
C22—C21—P3	120.0 (3)	C71—C66—C67	118.3 (6)
C23—C22—C21	120.1 (3)	C71—C66—C65	120.8 (6)
C23—C22—H22	119.9	C67—C66—C65	120.9 (6)
C21—C22—H22	119.9	C68—C67—C66	121.7 (7)
C24—C23—C22	121.0 (4)	C68—C67—H67	119.1
C24—C23—H23	119.5	C66—C67—H67	119.1
C22—C23—H23	119.5	C69—C68—C67	117.0 (7)
C23—C24—C25	119.5 (4)	C69—C68—H68	121.5
C23—C24—H24	120.2	C67—C68—H68	121.5
C25—C24—H24	120.2	C68—C69—C70	122.0 (7)
C24—C25—C26	120.6 (4)	C68—C69—H69	119.0
C24—C25—H25	119.7	C70—C69—H69	119.0
C26—C25—H25	119.7	C71—C70—C69	119.4 (6)
C25—C26—C21	120.4 (4)	C71—C70—H70	120.3
C25—C26—H26	119.8	C69—C70—H70	120.3
C21—C26—H26	119.8	C70—C71—C66	121.6 (6)
C28—C27—C32	119.3 (4)	C70—C71—H71	119.2
C28—C27—P3	122.2 (3)	C66—C71—H71	119.2
C32—C27—P3	118.5 (3)	Cl1—C72—Cl2	110.8 (3)
C29—C28—C27	119.9 (4)	Cl1—C72—H72A	109.5
C29—C28—H28	120.0	Cl2—C72—H72A	109.5
C27—C28—H28	120.0	Cl1—C72—H72B	109.5
C30—C29—C28	121.2 (5)	Cl2—C72—H72B	109.5
C30—C29—H29	119.4	H72A—C72—H72B	108.1
			
Pt1—C1—C2—C12	−17.8 (4)	Pt1—P2—C33—C34	25.8 (3)
Fe1—C1—C2—C12	48.8 (3)	C39—P2—C33—C38	71.6 (3)
Pt1—C1—C2—C3	171.6 (3)	C45—P2—C33—C38	−36.5 (3)
Fe1—C1—C2—C3	−121.8 (3)	Pt1—P2—C33—C38	−156.0 (3)
Pt1—C1—C2—Fe1	−66.57 (17)	C38—C33—C34—C35	−0.9 (5)
C1—C2—C3—C4	50.4 (5)	P2—C33—C34—C35	177.4 (3)
C12—C2—C3—C4	−118.9 (4)	C33—C34—C35—C36	0.9 (6)
Fe1—C2—C3—C4	−39.8 (5)	C34—C35—C36—C37	−0.8 (6)
C1—C2—C3—C10	−126.4 (4)	C35—C36—C37—C38	0.8 (6)
C12—C2—C3—C10	64.3 (5)	C36—C37—C38—C33	−0.9 (6)
Fe1—C2—C3—C10	143.4 (3)	C34—C33—C38—C37	1.0 (5)
C10—C3—C4—C5	0.9 (6)	P2—C33—C38—C37	−177.3 (3)
C2—C3—C4—C5	−176.0 (4)	C33—P2—C39—C40	3.1 (3)
C3—C4—C5—C7	−0.8 (6)	C45—P2—C39—C40	115.3 (3)
C3—C4—C5—C6	179.5 (4)	Pt1—P2—C39—C40	−133.9 (3)
C4—C5—C7—C9	0.0 (6)	C33—P2—C39—C44	177.3 (3)
C6—C5—C7—C9	179.7 (4)	C45—P2—C39—C44	−70.5 (3)
C4—C5—C7—C8	−179.3 (4)	Pt1—P2—C39—C44	40.3 (3)
C6—C5—C7—C8	0.4 (6)	C44—C39—C40—C41	0.0 (6)
C5—C7—C9—C10	0.7 (6)	P2—C39—C40—C41	174.2 (3)
C8—C7—C9—C10	−180.0 (4)	C39—C40—C41—C42	0.1 (6)
C7—C9—C10—C3	−0.6 (6)	C40—C41—C42—C43	0.1 (6)
C7—C9—C10—C11	175.8 (4)	C41—C42—C43—C44	−0.5 (6)
C4—C3—C10—C9	−0.2 (5)	C42—C43—C44—C39	0.6 (6)
C2—C3—C10—C9	176.7 (3)	C40—C39—C44—C43	−0.4 (5)
C4—C3—C10—C11	−176.5 (4)	P2—C39—C44—C43	−174.9 (3)
C2—C3—C10—C11	0.4 (6)	C46—P1—C45—P2	179.53 (17)
C1—C2—C12—O1	135.7 (5)	C52—P1—C45—P2	74.8 (2)
C3—C2—C12—O1	−53.8 (7)	Fe1—P1—C45—P2	−56.55 (18)
Fe1—C2—C12—O1	−171.1 (5)	C33—P2—C45—P1	−87.49 (19)
C1—C2—C12—Fe1	−53.2 (3)	C39—P2—C45—P1	163.46 (17)
C3—C2—C12—Fe1	117.2 (3)	Pt1—P2—C45—P1	43.94 (17)
C27—P3—C15—C16	96.0 (3)	C45—P1—C46—C47	38.0 (3)
C21—P3—C15—C16	−155.9 (3)	C52—P1—C46—C47	141.6 (3)
Pt1—P3—C15—C16	−25.2 (3)	Fe1—P1—C46—C47	−86.2 (3)
C27—P3—C15—C20	−83.6 (3)	C45—P1—C46—C51	−146.9 (3)
C21—P3—C15—C20	24.5 (4)	C52—P1—C46—C51	−43.3 (3)
Pt1—P3—C15—C20	155.2 (3)	Fe1—P1—C46—C51	88.9 (3)
C20—C15—C16—C17	0.5 (5)	C51—C46—C47—C48	1.7 (6)
P3—C15—C16—C17	−179.1 (3)	P1—C46—C47—C48	176.9 (3)
C15—C16—C17—C18	−0.8 (6)	C46—C47—C48—C49	−0.2 (7)
C16—C17—C18—C19	−0.4 (6)	C47—C48—C49—C50	−0.8 (7)
C17—C18—C19—C20	1.8 (6)	C48—C49—C50—C51	0.3 (6)
C18—C19—C20—C15	−2.1 (6)	C47—C46—C51—C50	−2.2 (6)
C16—C15—C20—C19	0.9 (6)	P1—C46—C51—C50	−177.5 (3)
P3—C15—C20—C19	−179.4 (3)	C49—C50—C51—C46	1.2 (6)
C15—P3—C21—C26	−136.5 (3)	C46—P1—C52—C53	150.9 (3)
C27—P3—C21—C26	−26.2 (3)	C45—P1—C52—C53	−103.8 (3)
Pt1—P3—C21—C26	95.1 (3)	Fe1—P1—C52—C53	23.1 (3)
C15—P3—C21—C22	50.2 (3)	C46—P1—C52—C57	−34.5 (3)
C27—P3—C21—C22	160.5 (3)	C45—P1—C52—C57	70.8 (3)
Pt1—P3—C21—C22	−78.1 (3)	Fe1—P1—C52—C57	−162.3 (2)
C26—C21—C22—C23	−0.4 (5)	C57—C52—C53—C54	1.5 (5)
P3—C21—C22—C23	173.1 (3)	P1—C52—C53—C54	176.2 (3)
C21—C22—C23—C24	0.6 (6)	C52—C53—C54—C55	0.6 (6)
C22—C23—C24—C25	−0.7 (6)	C53—C54—C55—C56	−1.7 (6)
C23—C24—C25—C26	0.7 (6)	C54—C55—C56—C57	0.8 (6)
C24—C25—C26—C21	−0.5 (6)	C55—C56—C57—C52	1.4 (6)
C22—C21—C26—C25	0.4 (5)	C53—C52—C57—C56	−2.5 (5)
P3—C21—C26—C25	−173.0 (3)	P1—C52—C57—C56	−177.2 (3)
C15—P3—C27—C28	29.2 (4)	C64—C59—C60—C61	−0.5 (7)
C21—P3—C27—C28	−79.8 (3)	C58—C59—C60—C61	178.2 (4)
Pt1—P3—C27—C28	153.3 (3)	C59—C60—C61—C62	−0.4 (7)
C15—P3—C27—C32	−151.3 (3)	C60—C61—C62—C63	1.0 (8)
C21—P3—C27—C32	99.6 (3)	C61—C62—C63—C64	−0.8 (8)
Pt1—P3—C27—C32	−27.3 (3)	C60—C59—C64—C63	0.7 (7)
C32—C27—C28—C29	0.9 (6)	C58—C59—C64—C63	−177.9 (4)
P3—C27—C28—C29	−179.7 (3)	C62—C63—C64—C59	−0.1 (8)
C27—C28—C29—C30	0.3 (7)	C71—C66—C67—C68	−1.8 (8)
C28—C29—C30—C31	−1.5 (7)	C65—C66—C67—C68	178.6 (5)
C29—C30—C31—C32	1.6 (7)	C66—C67—C68—C69	0.6 (9)
C28—C27—C32—C31	−0.7 (6)	C67—C68—C69—C70	1.2 (9)
P3—C27—C32—C31	179.8 (3)	C68—C69—C70—C71	−1.7 (8)
C30—C31—C32—C27	−0.5 (6)	C69—C70—C71—C66	0.3 (7)
C39—P2—C33—C34	−106.7 (3)	C67—C66—C71—C70	1.4 (7)
C45—P2—C33—C34	145.2 (3)	C65—C66—C71—C70	−179.0 (4)

### Hydrogen-bond geometry (Å, º)

*Cg*3, *Cg*6, *Cg*8, *Cg*9 and *Cg*10 are the centroids of the C21–C26, C39–C44, C52–C57, C59–C64 and C66–C71 rings, respectively.

**Table:**

*D*—H···*A*	*D*—H	H···*A*	*D*···*A*	*D*—H···*A*
C11—H11*A*···O1	0.96	2.36	3.193 (4)	145
C31—H31···O1^i^	0.93	2.55	3.370 (5)	147
C41—H41···O2^ii^	0.93	2.49	3.202 (5)	134
C48—H48···O3^iii^	0.93	2.46	3.325 (5)	154
C11—H11*B*···*Cg*9^iv^	0.96	2.80	3.719 (4)	160
C22—H22···*Cg*6	0.93	2.80	3.597 (3)	145
C34—H34···*Cg*3	0.93	2.98	3.519 (4)	118
C38—H38···*Cg*10	0.93	2.82	3.694 (4)	156
C60—H60···*Cg*8	0.93	2.81	3.543 (5)	137

Symmetry codes: (i) *x*+1, *y*, *z*; (ii) −*x*+1/2, *y*+1/2, −*z*+3/2; (iii) *x*−1, *y*, *z*; (iv) *x*−1/2, −*y*+1/2, *z*−1/2.
